# Coastal Wild Grapevine Accession (*Vitis vinifera* L. ssp. *sylvestris*) Shows Distinct Late and Early Transcriptome Changes under Salt Stress in Comparison to Commercial Rootstock Richter 110

**DOI:** 10.3390/plants11202688

**Published:** 2022-10-12

**Authors:** David Carrasco, Andres Zhou-Tsang, Alberto Rodriguez-Izquierdo, Rafael Ocete, María Angeles Revilla, Rosa Arroyo-García

**Affiliations:** 1 CSIC-INIA(CBGP) Centro de Biotecnología y Genómica de Plantas, UPM-INIA, Parque Científico y Tecnológico de la UPM Campus de Montegancedo, CtraM-40, Km 38, Pozuelo de Alarcón, 28223 Madrid, Spain; 2 Waite Research Institute, The School of Agriculture, Food and Wine, Faculty of Sciences, Engineering and Technology, The University of Adelaide, Glen Osmond, SA 5064, Australia; 3 ARC Industrial Transformation Training Centre for Innovative Wine Production, Waite Research Institute, Glen Osmond, SA 5064, Australia; 4 Laboratorio Entomología Aplicada, Universidad de Sevilla, Avenida Reina Mercedes 6, 41012 Sevilla, Spain; 5 Departamento Biología de Organismos y Sistemas, Facultad de Biología, Universidad de Oviedo, 33071 Oviedo, Spain

**Keywords:** wild grapevine accession, rootstock, salinity, salt tolerance, transcriptomic analysis, gene ontology

## Abstract

Increase in soil salinity, driven by climate change, is a widespread constrain for viticulture across several regions, including the Mediterranean basin. The implementation of salt-tolerant varieties is sought after to reduce the negative impact of salinity in grape production. An accession of wild grapevine (*Vitis vinifera* L. ssp. *sylvestris*), named AS1B, found on the coastline of Asturias (Spain), could be of interest toward the achievement of salt-tolerant varieties, as it demonstrated the ability to survive and grow under high levels of salinity. In the present study, AS1B is compared against widely cultivated commercial rootstock Richter 110, regarding their survival capabilities, and transcriptomic profiles analysis allowed us to identify the genes by employing RNA-seq and gene ontology analyses under increasing salinity and validate (via RT-qPCR) seven salinity-stress-induced genes. The results suggest contrasting transcriptomic responses between AS1B and Richter 110. AS1B is more responsive to a milder increase in salinity and builds up specific mechanisms of tolerance over a sustained salt stress, while Richter 110 maintains a constitutive expression until high and prolonged saline inputs, when it mainly shows responses to osmotic stress. The genetic basis of AS1B’s strategy to confront salinity could be valuable in cultivar breeding programs, to expand the current range of salt-tolerant rootstocks, aiming to improve the adaptation of viticulture against climate change.

## 1. Introduction

Grapevine (*Vitis vinifera L.*) is one of the most extended crops in the world, especially in the Mediterranean area. The value of this crop is represented across agronomical, food, biotechnological, and commercial sectors, among others. The largest grape-growing region dedicated to wine production in the world is Spain, possessing an area of more than 950,000 ha; hence, grapevine has a particularly special importance in Spain, with great economic representation (EUR 1–1.5 billion, 10% of total agricultural production), social value represented by over 400,000 producers (contributing to promote rural population fixation), and great representation in the environment and landscapes [1]. Given the importance of the grapevine to society, continuous efforts in scientific research and industrial innovation are required to preserve grape production against biotic and abiotic stresses, especially in the case of climate change.

The health status of grapevines, and consequently their grape production, can be affected by environmental challenges such as salinity, water scarcity, and extreme temperatures, which are some of the effects of climate change [2,3]. Salinity in soils represents an extended problem in Mediterranean agriculture [4,5], and it mainly aggravates due to recursive groundwater irrigation, as the mineral content dissolved in groundwater accumulates in soil after evaporation [6]. Both the grape and wine sectors are affected by salinity in terms of yield loss and alteration of juice taste [7,8], as grapevines are relatively sensitive to salinity [9]. Commercially available rootstocks such as Ramsey (*Vitis × champinii*, 1103 Paulsen (*Vitis berlandieri* × *Vitis rupestris*), Freedom (*Vitis champinii* × (*V. solonis* × *V. riparia*), or 140 Ruggeri (*Vitis berlandieri* × *Vitis rupestris*) have demonstrated the ability to confer a range of protection against salinity to the grafted fruiting scion [8,10,11,12]; however, the current range of salt-excluding rootstocks is limited and may be insufficient for future degrees of soil salinization [13,14]. Therefore, research into and the development of new salt-tolerant varieties that, additionally, may present additional agronomic traits for disease resistance and environmental adaptations, will be essential to sustain and improve grape production against the expanding threat of soil salinization through climate change.

In the Mediterranean area, local wild-grapevine accessions (*Vitis vinifera* subsp. *sylvestris*) have been proposed as a resource for breeding due to their naturally selected abiotic and biotic tolerances [15]. The study of salt-tolerant wild populations could open multiple possibilities to expand the knowledge on the mechanisms of salt tolerance in grapevines [16,17] and, thus, create possibilities for developing salt-tolerant varieties. Furthermore, research on wild populations can promote the value of natural reservoirs as well as the importance of their preservation [18].

In the present study, we propose that a good candidate to envisage the potential of wild populations for salt tolerance is AS1B, a grapevine accession originally sampled from the coastal areas of Asturias (Spain). It can be expected that a plant genotype inhabiting saline coastlines would develop biological mechanisms to thrive under salinity, and such is the case of AS1B [19]. In a preliminary study with different wild-grapevine accessions, AS1B showed a higher survival capability under salinity compared to the commercial rootstock Richter 110, which is a widely cultivated variety for semi-arid environments [20]. Therefore, the present study aims to detect the root transcriptomic patterns in AS1B when facing different NaCl concentrations, over different exposure times, and elucidate how these patterns compare to a less salt-tolerant commercial rootstock such as Richter 110, in order to understand the genetic components for salt tolerance in AS1B.

Our findings could help to design new strategies to adapt grape production to climate change, based on the traits that wild-grapevine populations possess as part of their natural diversity. Moreover, the present study reveals transcriptomic profiles of each genotype and identifies new potential candidate genes involved in the response against salinity stress, which would set the path for a grapevine-specific functional characterization of these genes.

## 2. Results

### 2.1. Pre-Harvest Mortality Rates in AS1B and Richter 110

When irrigated with increased salinity conditions, the total pre-harvest mortality rates for 85 mM NaCl after 7 days were 25.14% in R110 and 3.92% in AS1B. For 120 mM NaCl after 1 day, mortality rates reached 100% in Richter 110 and remained at 3.92% in AS1B. It was not until AS1B was irrigated with 120 mM NaCl, after 14 days, when the wild genotype complete mortality. This demonstrated that AS1B can endure salinity better than Richter 110.

### 2.2. Differential Gene Expression between AS1B and Ricther 110 under Salt-Stress Conditions

**Figure 1:**
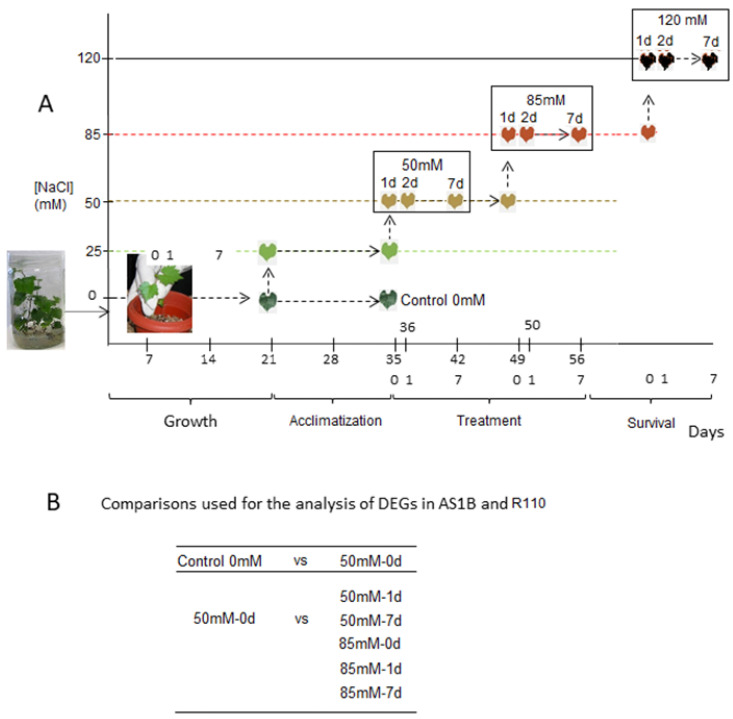
Experimental design and time course of the salt treatments on AS1B and R110 accessions. (**A**) Plants were irrigated with 200 mL of Hoagland’s solution every 2 days for 3 weeks, for the initial growth of the plants. Then, plants were treated with Hoagland solution with the addition of 25 mM NaCl for adaptation phase for 2 weeks, and after that the conditions were created with an increase of 50 mM to 120 mM NaCl. (**B**) Treatment conditions selected for differential expression analysis.

**Figure 2:**
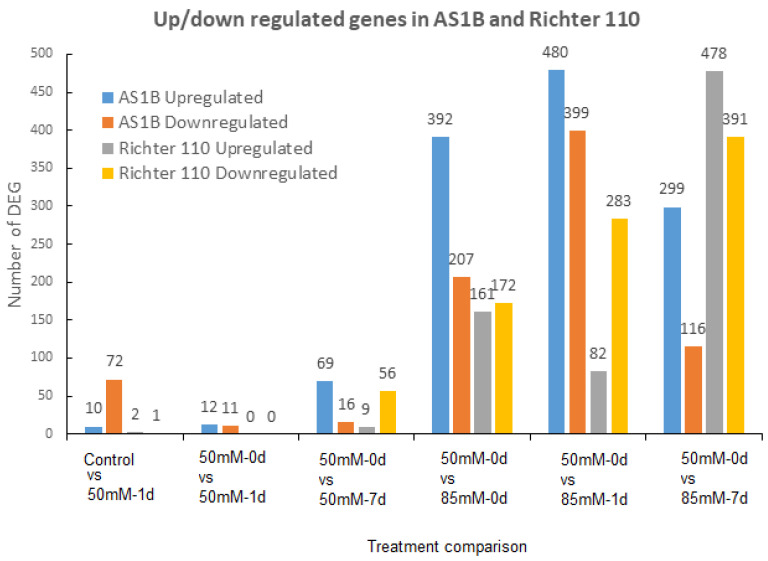
Number of DEGs detected in AS1B and Richter 110 through all the conditions.

In our transcriptomic assay under salt stress conditions (Figure 1A), we have been able to identify 25.390 ± 421 transcripts from the control group and 25.526 ± 227 transcripts from the different treatment conditions (Supplementary Data S1), with similar coverage for both genotypes (85.98% ± 2.86 for AS1B and 79.8% ± 1.95 for R110). Across all the RNAseq comparisons (Figure 1B), we have found a total of 3718 differentially expressed genes (DEGs) which includes 1994 upregulated and 1724 downregulated transcripts (Figure 2 and Supplementary Data S2).

The first transcriptome analyses comparison between Control vs. 50 mM0 d showed an early response in AS1B compared with R110 (Figure 2) with 82 DEG (72 downregulated and 10 upregulated) in AS1B and only 3 DEGs in R110, indicating that the AS1B genotype can rapidly respond to an increase in saline concentration, while R110 mostly maintains its constitutive expression. When we analyzed the effect of 50 mM NaCl over one day (comparing 50 mM-0 d vs. 50 mM-1 d), we observed very few differences (23 DEGs for AS1B and no DEG for R110), indicating that there is still no response to the new conditions and that only AS1B has the capacity for a fast response to new stress conditions. In the following comparison (50 mM-0 d vs. 50 mM-7 d), we analyze the effect at 7 days of exposure to 50 mM NaCl, and we observe a first response of the upregulation of 69 genes in AS1B, while in R110 there is mainly the downregulation of 56 genes, similar to the downregulation observed in AS1B at 1 h after treatment, indicating a delayed response to a change in salinity conditions.

In the following three comparisons, the effect of the increase in salt concentration between 50 mM to 85 mM NaCl was analyzed. First, we compared the vines exposed to 50 mM NaCl for 1 h (50 mM-0 d) against those exposed to 85 mM NaCl for 1 h (85 mM-0 d); here, we observed a significant increase in the DEGs in both genotypes (Figure 2). The main difference between the genotypes is that AS1B presented more DEGs than R110; here, AS1B was counted with twice the number of upregulated genes compared to downregulated genes, while R110 displayed a similar number of upregulated and downregulated genes. These results suggest that AS1B produces a more intense and faster transcriptomic response than R110 to an increase in saline conditions. In the following comparison (50 mM-0 d vs. 85 mM-1 d), greater differences between the genotypes are observed. On the one hand, the DEG number has duplicated in AS1B compared to R110, and, in AS1B, there continues to be a greater number of upregulated than downregulated genes; while, in R110, there is a greater number of downregulated than upregulated genes. The results indicate again that AS1B has a higher number of genes that respond to a change in salinity than R110. In addition, R110 displays a higher number of downregulated than upregulated genes, suggesting a distinct adaptation to salinity, by decreasing the activity of the processes in which these genes could be involved (Figure 2). In the last analysis (50 mM-0 d vs. 85 mM-7 d), we studied the effects of salinity on the vines after 7 days of exposure to 85 mM of NaCl. This comparison shows again greater gene expression response in AS1B in contrast to R110; in addition, for the first time, we observe that in both genotypes there is a higher number of upregulated genes than downregulated genes. It is also noted that the number of DEGs in AS1B is lower than in the previous comparison (50 mM-0 d vs. 85 mM-1 d), suggesting an attenuation in the response of the plants to salinity. On the other hand, in R110, a large increase in the number of DEGs is observed compared to previous comparisons, suggesting that this genotype has a slower response, which we had already observed in previous periods.

### 2.3. Gene Ontology Analysis

**Figure 3:**
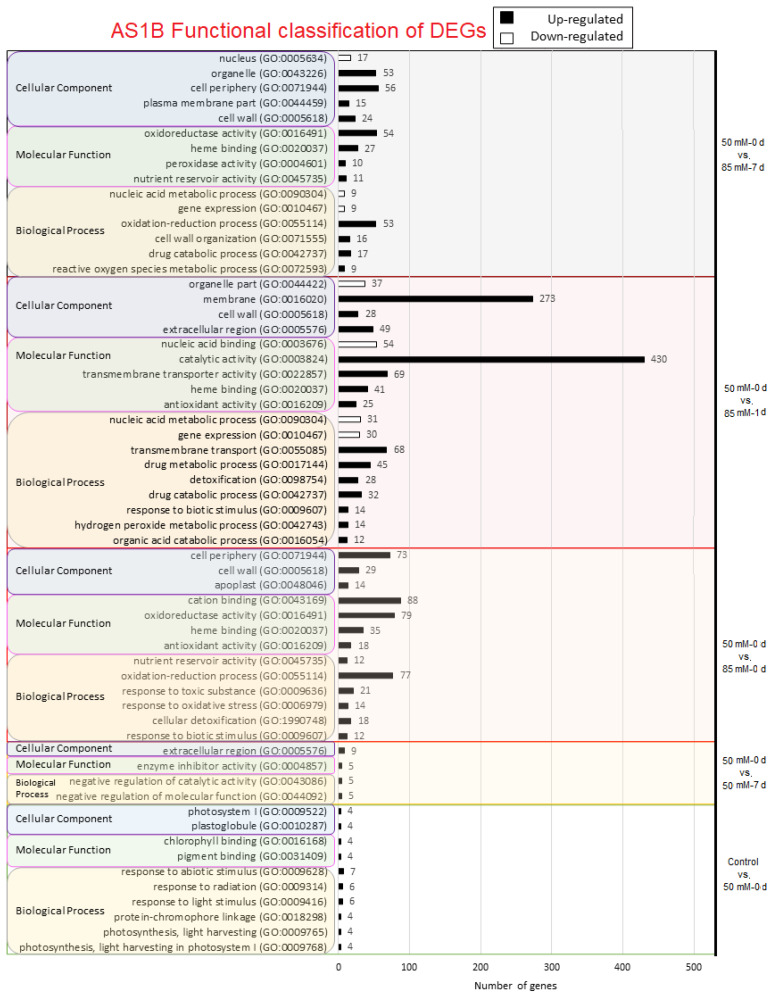
Panther output of GO results obtained in DEG analysis in AS1B under different comparisons: Control vs. 50 mM-0 d (in green region), 50 mM-0 d vs. 50 mM-1 d (brown region), 50 mM-0 d vs. 50 mM-7 d (yellow region), 50 mM-0 d vs. 85 mM-0 d (red region), and 50 mM-0 d vs. 85 mM-7 d (gray region). Number of GO detected are expressed in upregulated (black bars) and downregulated (white bars) GO functions. For each comparison, 3 GO levels are shown: biological process (gray-outlined yellow box), molecular function (pink-outlined green box regions), and cellular component (violet-outlined gray box).

**Figure 4:**
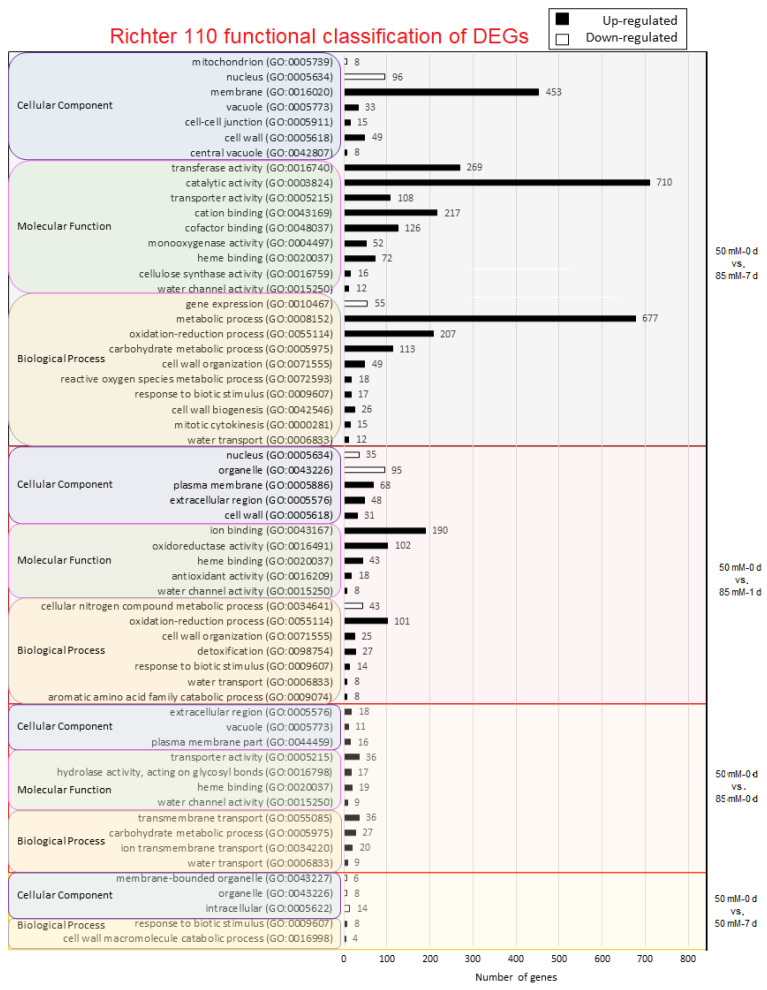
Panther output of GO results obtained in DEG analysis in Richter 110 under different comparisons: Control vs. 50 mM-0 d (green region), 50 mM-0 d vs. 50 mM-1 d (brown region), 50 mM-0 d vs. 50 mM-7 d (yellow region), 50 mM-0 d vs. 85 mM-0 d (red region), and 50 mM-0 d vs. 85 mM-7 d (gray region). Number of GO detected are expressed in upregulated (black bars) and downregulated (white bars) GO functions. For each comparison, 3 GO levels are shown: biological process (gray-outlined yellow box), molecular function (pink-outlined green box), and cellular component (violet-outlined gray box).

To determine the possible functions that can be attributed to the DEGs produced by salinity, we did gene ontology (GO) analysis. GO enrichment for AS1B is indicated in Figure 3 and for R110 in Figure 4. In the first comparison, between control vs. 50 mM-0 d, only the upregulated genes of the AS1B genotype presented GO enrichment, mainly in response to abiotic stimulus, photosynthesis, and chlorophyll binding; however, considering the analysis being performed on root tissues, functions will need to be contextualized (Figure 3). No GO results were found at 50 mM-0 d vs. 50 mM-1 d, but at 50 mM-0 d vs. 50 mM-7 d, AS1B showed enrichment in the biological process (BP) related to the negative regulation of catalytic activity and molecular function, whereas, in R110 BP, there is an enriched in response to biotic stimulus and the cell wall macromolecule catabolic process. For the next comparison (50 mM-0 d vs. 85 mM-0 d), all the GO enrichments are different between the two genotypes. In both cases, only upregulated genes showed GO enrichment, and the number of enriched genes is higher in AS1B. The main enrichments in AS1B involved BP for oxidation-reduction, response to oxidative stress, and cellular detoxication; molecular functions (MF) for cation binding and oxidoreductase activity; and cellular component (CC) activities for the cell periphery, cell wall, and apoplast. On the other hand, in R110, there is an enrichment in BP as the transmembrane ion transport and water transport; in MF transporter activity and water channel activity; and in the CC vacuole, plasma membrane, mitochondria, cell wall, nucleus, and cell junction.

For the comparison of 50 mM-0 d vs. 85 mM-1 d, there is also a higher enrichment of GO elements in AS1B than in R110. Both genotypes share enrichment in detoxification and response to biotic stimulus (within the BP category); in MF, they share heme binding and antioxidant activity; and, in CC, they share organelle, cell wall, and extracellular regions. When it comes to the differences, we can highlight BP enrichment in AS1B in transmembrane transport and the drug metabolic process (for upregulated genes), in gene expression and the nucleic acid metabolic process (for downregulated genes), and in MF catalytic and transmembrane transporter activities. Meanwhile, R110 stands out in BP-oxidation-reduction and water transport processes and also in MF-ion-binding and oxidoreductase activities.

In the last comparison (50 mM-0 d vs. 85 mM-7 d), R110 is now the genotype with higher enrichments, suggesting its highest response to salt occurs after a longer exposure and under higher concentrations than AS1B. GO enrichment in AS1B highlights oxidoreductase activity (MF) and organelle and cell periphery (CC). Nevertheless, the most remarkable results in this comparison are the enrichments in GO in R110, as it stands out in the BP-metabolic process, oxidation-reduction process, and carbohydrate-metabolic process; in relation to MF, transferase, catalytic, and transporter activity and cation binding stand out.

DEA and GO indicate differences in the transcriptional strategies that AS1B and Richter 110 have developed to withstand salinity. GO analysis showed that, during the 50 mM conditions, AS1B presented a different functional profile than R110, increasing the number of DEGs related to oxidative stress, cellular detoxification, and cation binding (Figure 3 and Figure 4). Whereas the strategy of AS1B seemed to be inclined toward detoxification and reduction in ROS from cellular stresses, R110 mainly increased the number of DEGs for water channels in the vacuole and cell membrane (Figure 3 and Figure 4). As salt concentration in the treatment advances toward 85 mM, differences between the strategies of AS1B and R110 became more evident. GO results in AS1B showed more expression in detoxification to reduce ROS and transmembrane transporters (Figure 3), while R110 increased the gene expression of the genes related to the water- and fluid-transportation process, which are also involved in the plasma membrane and extracellular region (Figure 4). In addition, R110 showed more genes involved in cation binding and oxidoreductase activity. In the last comparison (50 mM-0 d vs. 85 mM-7 d), R110 seemed to increase the amount of DEGs involved in ion detoxification, in addition to catalytic activity and the cell membrane (Figure 4).

### 2.4. Consistent DEGs in AS1B and Richter 110 across Different Treatment Conditions

**Figure 5:**
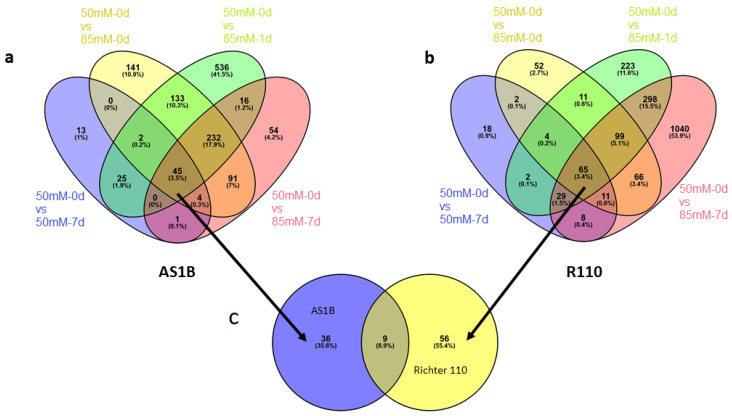
Venn diagram representation of DEGs under different salinity conditions (from 50 mM-0 d vs. 50 mM-7 d to 50 mM-0 d vs. 85 mM-7 d) for AS1B (**a**) and Richter 110 (**b**). Number of overlapped and non-overlapped areas that represent DEGs through the different conditions of salinity stress, with their equivalent percentages from the total amount of DEGs retrieved by consensus between NOISeq and DESeq2 pipelines. (**c**) Venn diagram representation of genes showing continuous differential expression across all conditions. Overall, 36 unique DEGs from AS1B, 56 DEGs from Richter 110, and 9 DEGs shared in common between genotypes (in total, 45 and 65, respectively, as seen in the core areas of (**a**,**b**)), with their equivalent percentages from the total combined amount of DEGs between genotypes.

**Figure 6:**
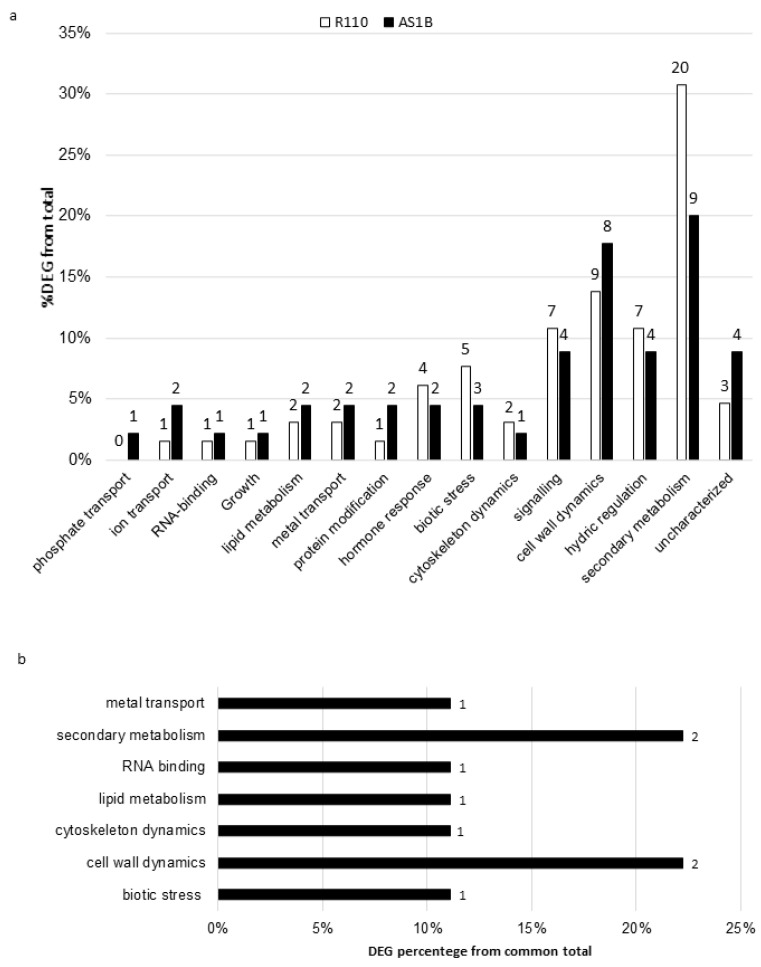
Functional distribution of AS1B and Richter 110 DEGs: DEGs by each accession (**a**); DEGs in common for both AS1B and Richter 110 (**b**). DEG percentages were calculated from the total amount of DEGs in each accession (45 DEGs for AS1B and 65 DEGs for Richter 110, with 9 DEGs among them). Numbers represented in the figure above and beside them represent the number of DEGs founded for each functional group.

Venn diagram representation of DEGs across different comparisons (50 mM-0 d vs. 50 mM-7 d and 50 mM-0 d vs. 85 mM-0 d, 85 mM-1 d, and 85 mM-7 d) in AS1B and R110 (Figure 5) showed a core group of genes persistently expressed across the different salinity-stress conditions (overlapping areas of every condition shown in Figure 5), consisting of 45 DEGs in AS1B (Figure 5a) and 65 DEGs in R110 (Figure 5b), with both genotypes sharing 9 DEGs (Figure 4). Given their recurrence across salt concentrations and times, these groups of DEGs could potentially represent important resources from both varieties in their response against salt inputs. These groups of genes were annotated in GO and categorized in functional groups (Figure 6). Some of them were phosphate transport, ion transport, RNA-binding, growth, or hydric regulation. Results suggest that AS1B favored ion transport and protein modification, while R110 was more addressed to biotic stress, signaling, hormone response, hydric regulation, and secondary metabolism (Figure 6a). However, AS1B and R110 have similar rates in cell wall dynamics. Furthermore, the functional characterization for DEGs genes in both accessions shows their prominence in cytoskeleton dynamics and secondary metabolism (Figure 6b).

From the set of DEGs with homology to coding sequences, seven genes were selected for further validation via RT-qPCR (Supplementary Data S6). Selected genes were chosen on the basis of our results, associating them with salinity stress response. The differential display analysis was confirmed for the seven genes selected by RT-qPCR and regression analysis of significantly regulated RNAseq LFC and qPCR LFC values. (Supplementary Figures S4 and S5).

## 3. Materials and Methods

### 3.1. Preparation of Plant Material and Salt Treatments

Experiments were performed using a total of 33 clonal plants from AS1B and another 33 from Richter 110. Clonal plants were obtained by in vitro propagation from stem cuttings and, subsequently, grown on perlite substrate in 5 L pots for 3 weeks. During the initial growth stage, every individual plant was irrigated with 200 mL of Hoagland’s solution [21] every 2 days.

Salt treatments were designed with increasing concentrations and time (Figure 1), to simulate natural conditions where, generally, the increase in NaCl occurs gradually in the environment [22]. Treatment groups were watered using a salinized Hoagland solution, through the addition of 25 mM NaCl for 2 weeks, to induce their acclimatization to a slightly saline environment, to avoid osmotic shock when switching to the treatment with higher concentrations (Figure 1) [23]. Next, salt treatments were implemented with 50 mM NaCl in the irrigation media for another two weeks and then increased to 85 mM for two weeks (Figure 1A). During the treatment stages, roots were harvested at three time points after their first irrigation: 1 h (0 days), 1 day, and 7 days. Immediately after harvesting, roots were transferred into liquid nitrogen and stored at −80 °C. Finally, to observe the survival limits for both genotypes, an additional step of irrigation with 120 mM of NaCl was applied for a period of another 2 weeks. Meanwhile, control groups of plants continued to receive irrigation with base Hoagland’s solution, and harvesting times were the same as those of the salt-treated plants.

### 3.2. RNA Extraction and Sequencing

Frozen roots were employed for isolation of RNA, following the procedure described in Reid et al. [24]. RNA was not extracted from samples at 120 mM NaCl, since the differences in mortality rates led to unpaired samples sizes between AS1B and Richter 110, which would affect their transcriptomic comparison. Isolates of RNA were sequenced at Novogene (UK) Company Limited, Cambridge, UK, using Illumina Hiseq 2000 systems for paired-end sequencing and Strong Specific library. Over 33 million sequences per sample were produced, with a length of 150 bp.

### 3.3. Differential Expression Analysis

RNA-Seq data from both treated samples and control samples (Figure 1B) were loaded into the computer cluster CESGA (Centro de Supercomputación de Galicia, Santiago de Compostela, Spain) for the following bioinformatic analyses. The quality of sequences was checked using FastQC v0.11.7 [25]. Trimming sequences were performed using Trimmomatic 0.38, and trimmed data were aligned using HISAT2 v2.1.0 [26] to the grapevine reference genome and transcriptome 12X V3 of accession PN40024 (slight genetic differences were expected between the PN40024 reference genome and the accessions used in this study [27]). Gene annotation codes from reference genome, such as RefSeq from NCBI, were used for each gene. Gene expression was quantified using Stringtie v1.3.5 [28]; differential expression gene analyses were performed using NOISeq v2.26.0 [29] and DESeq2 v3.9 [30] in R 3.6 environment [31] and Bioconductor v3.9 software pipelines. Resulting differentially expressed genes (DEGs) were selected by consensus among the outputs of NOISeq and DESeq2 runs [32]; this consensus was calculated by using prepDE.py script in Python v3.0 environment to adapt counts of genes by both differential expression analysis tools. A range of −2 ≥ log2fold change ≥ 2 was established to accept the differential expression in genes, within *p* value < 0.05.

### 3.4. Gene Ontology Analysis

Gene ontology for DEGs was determined using Blast2GO software (version 5.2.5, BioBam Bioinformatics, Valencia, Spain), based on proteins’ annotations from the INTERPRO database [32]. Uncharacterized proteins in v1 annotation of PN40024 were subject to the detection of possible homologies using NCBI BlastP alignment and MPI HHMER toolkit [33]. The distribution of DEGs into functional categories in control and treatment conditions were assigned into seven functional categories (cellular process, metabolism, regulation, response to stimulus, signaling, transport, and unknown) according to GrapeGen 12x_v2.1 annotation (http://genomes.cribi.unipd.it/grape/) (accessed on 11 March 2019).

### 3.5. Gene Validation: RT-qPCR and Linear Regression Analyse

For verification of RNA-seq results, we performed RT-qPCR analysis of the expression of seven genes (Vitvi17g00530; Vitvi02g00529; Vitvi01g01165; Vitvi06g00412; Vitvi08g01174; Vitvi02g01406; Vitvi02g01405). RNA was extracted as described above, and cDNA was synthesized using XLT cDNA superMix (Quantabio, Beverly, MA, USA) kit. Quantitative real-time PCR (qPCR) reactions were performed using LightCycler 480 SYBR Green I Master (ROCHE, Basel, Switzerland), in accordance with the manufacturer manual. The relative expression was calculated using the 2-DDCt method, using GAPDH and actin as reference genes for normalization. Finally, we have completed linear regression analysis using the log fold change values (LFC) with the RNAseq significant regulated transcripts vs. LFC obtained by qPCR and R-squared measure; lineal model was calculated by Microsoft Excel.

## 4. Discussion

### 4.1. Focusing the Comparison of Known Salt Damages and Mechanisms Tolerance in Grapevine

Transcriptomic datasets are generally complex in details, and the results in the present study are no exception. Two organisms of different species with an evolutionary divergence of 11 million years [34] are being compared here. Moreover, additional complexity comes from an experimental design that involves multiple stages of both salt concentration and time. Therefore, in order to scrutinize the relevant differences to salt tolerance, we are guided by the current knowledge available on the biological mechanisms that could be relevant to salt tolerance in grapevine; yet, this knowledge is still limited, so less specific information is often inferred from other plant species such as Arabidopsis [14].

In the physiological context, excessive salinity produces in grapevine a reduction in CO_2_ fixation and water potential, resulting in decreased shoot growth [35] and, consequently, lower yields in fruit production and quality [12,36]. Negative attributes can be perceived from wines made with salt-affected berries, such as an excessive salty and soapy taste [7,8]. During winemaking, the juice-fermentation process can be disrupted by slower yeast proliferation, leading to unfinished wine [37]. In terms of molecular biology, a broad range of interactions are involved in plant cells when facing salt damage: osmotic stress produced by high concentration of salts causes an inhibition of water intake in plants, impacting their growth and development. As plants are exposed to saline environments, inorganic ions, especially Na^+^ and Cl^−^, can accumulate in tissues, leading to a state of toxicity in the cells, while causing nutritional and energetic imbalances [38,39]. In addition, an increase in ionic concentration in the cells is associated with lipid peroxidation events, causing disorganization in the cell plasmatic membrane [40]. These processes increase the production of reactive oxygen species (ROS) and facilitate metabolic dysfunction that could inhibit photosynthesis, reduce nutrient acquisition, and eventually contribute to cell death [39,41,42].

Some *Vitis* species have developed different strategies to endure high salinity concentrations. Ion exclusion and vacuolar sequestration take place mostly in the root cortex and pericycle cells, preventing excessive uptake to more sensitive aerial parts, where chloroplast damage can happen [43,44,45,46]. The osmotic gradient between soil and tissues can be compensated using organic solutes, with lower toxicity than ions, as osmolytes such as sucrose or alditols such as myo-inositol, thus reducing water loss in the cells [40]. Furthermore, water loss by transpiration can be reduced by closing the stomata and secreting cuticle wax [47], while also stimulating aquaporin production and activity [48,49]. Stomata closure comes, however, at the cost of a decrease in gas exchange, thus limiting carbon assimilation and tissue growth [50,51]. Secondary metabolism is enhanced to reduce the impact on ROS, increasing the production of anthocyanins and flavonoids, common ROS acceptors in grapevine [52,53,54]. Responses in grapevine under stress are mediated by complex hormonal pathways. Hormones like abscisic acid (ABA), jasmonic acid (JA), salicylic acid (SA), or ethylene produce phosphorylation cascades mediated by leucine-rich kinases and serine/threonine kinases, which modulate the metabolism in order to adapt the plant to stress conditions [55,56,57,58]. Therefore, detrimental effects in grapevine caused by salinity can be ameliorated by the implementation of rootstocks possessing the biological capabilities that allow them to prevent salt damage to the grafted scion and to themselves [59].

### 4.2. Distribution of Differentially Expressed Genes from Functional Categories in Both Accessions

In the present study, saline treatments involving multiple time points and concentrations of salt allowed for broader insights into the transcriptional responses against salinity from AS1B and R110 and, consequently, revealed not only the potential molecular mechanisms of salt tolerance but also how their implementation can change over different stress intensities. Therefore, this leads to the question of whether some mechanisms of salt tolerance in grapevine are influenced by the expression of candidate genes with a certain timing and intensity of stimulus. Other transcriptomic studies in grapevine involved a single concentration of salt for a single time length [60], or across different time lengths [61,62], which could effectively reveal the different genes of salt tolerance in grapevine and rootstocks, either constitutively expressed or differentially expressed. However, our study suggests that this information could be beneficially expanded by combining treatments with different concentrations of salt and different timings. Generally, different trends can be observed from the transcriptomic profiles from both accessions, which could help to understand the potential genetic and transcriptomic attributes to unveil AS1B’s higher capability for survival under high saline inputs: differential expression changes at early stages of treatment at times, a specific response, and a lower number of DEGs at the final stage of treatment possibly lead to energy-saving or optimized pathways (Figure 2, Figure 3 and Figure 4).

An early response from AS1B is reflected by the highest difference in the amount of DEGs being seen at the initial stages of salinity (Figure 2): in the first two cases (control vs. 50 mM-0 d; 50 mM-0 d vs. 50 mM-1 d), only AS1B showed a response, and we have found seven DEGs with a GO attributed to the response to abiotic stimulus (Figure 3); this might indicate a more sensitive response from AS1B in detecting low changes in salinity (Section 4.3). On the contrary, the first response for Richter 110 comes at 50 mM-0 d vs. 50 mM-7 d, which appears to not present DEGs related to abiotic stress and even showed some related to biotic stress. Under the same saline concentration, 50 mM NaCl, Henderson et al. [60] concluded that the salt-exclusion mechanisms in 140 Ruggeri (a close relative to R110 from the same parental background, but featuring higher salt tolerance) were not induced by salinity but, rather, seem to remain constitutively expressed, which could a similar case for R110. Similarly, constitutive gene expression profile was also shown in salt-tolerant variety Ramsey (*Vitis champinii*) against a saline treatment of 25 mM NaCl [63]. Given AS1B’s higher survival capability under salt stress, its responsive strategy could be an additional alternative to consider, in addition to known strategies in rootstocks showing constitutive expression.

Changes in the number of ion-transport-related DEGs begin later, at comparison 4 (50 mM-0 d vs. 85 mM-0 d) (Figure 3 and Figure 4), where AS1B shows up to 88 cation-binding DEGs, while R110 displays 20 ion-transport DEGs; it is unclear if this represents a difference in the expression of salt-exclusion-related genes. A clear example of early response is present in the categories related to oxidative stress defense (i.e., oxidoreductase activity, antioxidant activity, oxide reduction process, response to oxidative stress, and heme-binding), where AS1B begins displaying a higher expression at comparison 4 (50 mM-0 d vs. 85 mM-0 d), while R110 starts at comparison 5 (50 mM-0 d vs. 85 mM-1 d), with a higher number of DEGs compared to those of AS1B. Antioxidant activity is a key defense against ROS produced under saline conditions, which is a known mechanism in grapevine against salt stress [64,65], and higher production of antioxidative enzymes at lower salt concentrations was shown to be more beneficial against lipid peroxidation [65]; therefore, AS1B expressing antioxidant-related DEGs earlier than R110 could result in an advantage against the accumulation of ROS over time.

It is known that salinity can be constrain energy metabolism [38], not only through increased ATP consumption but also in terms of possible disruptions in mitochondrial respiration [63,66,67]. The GO categories “catalytic activity” and “metabolic process” can be associated with energy metabolism and the production and consumption of ATP, respectively [68]. The DEGs for catalytic activity are more abundant in AS1B at comparison 5 (50 mM-0 d vs. 85 mM-1 d) (Figure 3) and in R110 at comparison 6 (50 mM-0 d vs. 85 mM-7 d), together with metabolic-process-related DEGs (Figure 4). Therefore, a potential strategy from AS1B, involving an increase in ATP production at an earlier stage of salinity, could be more effective if at later stages this process is affected by mitochondrial salt stress. Prinsi et al. [69] suggested energetic metabolism as the main component of salt tolerance in the novel variety M4, in contrast to salt exclusion; even though this enhances survival, it would not be a suitable trait for a rootstock, as it might allow ion uptake to a vulnerable scion [12].

### 4.3. Key Functional Roles in DEGs Present in AS1B’s Early Response to the Initial Saline Input

Genes being differentially expressed by AS1B at the initial stages of treatment (control vs. 50 mM-0 d and 50 mM-0 d vs. 50 mM-1 d) can be important indicators of a higher sensitivity by AS1B to an increase in environmental salinity, especially when Richter 110 has not yet developed large changes in gene expression. A total of 82 DEGs are present in control vs. 50 mM-0 d, of which 57 DEGs possess their functional annotation in the grapevine PN40024 reference genome [27], and 25 DEGs remain uncharacterized (Supplementary Data S5). However, 45 of these DEGs were given a gene ontology category (Figure 3). Later, in 50 mM-0 d vs. 1 d 50 mM-0 d, 24 DEGs are shown for AS1B, from which 21 have been functionally annotated, while still none appear in Richter 110.

#### 4.3.1. Control vs. 50 mM-0 d

Two DEGs coding WRKY transcription factors are present here: Vitvi07g00421 and Vitvi07g00434. WRKY transcription factors are known to mediate responses to both biotic and abiotic stresses in a broad range of plant clades [70], and, in particular, some from members in *Vitis* have been able to confer tolerance to salinity and cold [71]. Another two DEGs coding kinase proteins can also be observed in this category: ser/thr kinase Vitvi07g01323 and adenylate kinase Vitvi14g01574. Another type of transcription factors are NAC domain proteins, from which Vitvi19g00270 appears as a DEG here. Some NAC domain proteins are known to be able to enhance salt tolerance in rice (*Oryza sativa*), tomato (*Lycopersicon solanum*), and kiwifruit (*Actinidia chinensis*) [72,73,74]. In other transcriptomic studies on *Vitis,* NAC domain proteins have been found to be differentially upregulated by salinity in tolerant cultivars such as 1103 Paulsen and Ramsey [58,75], which further supports the possibility of NAC being involved in salt tolerance; yet, the specific target of these transcription factors in grapevine remain uncharacterized.

Ser/threonine kinases are well-known to participate in salt stress responses in different species such as soybean (*Glycine soja*), wheat (*Tricum aestivum*), and Arabidopsis (*Arabidopsis thaliana*) [76,77,78,79], while adenylate kinase is associated with energetic metabolism, which, nonetheless, has been revealed to be upregulated in tolerant cultivars of soybean [80]. Additionally, another DEG in this category, Vitvi05g00461, codes for a putative membrane-associated kinase regulator. Likewise, a ser/thr phosphatase Vitvi13g01842 is present as a DEG, which would hypothetically complement the activity of the aforementioned kinases by reversing their phosphorylation activity [81].

Four DEGs coding for F-box proteins are listed here: Vitvi10g00837, Vitvi15g00832, Vitvi16g00957, and Vitvi17g00570. Some members of the F-box protein family have been associated with salt tolerance, such as F-box EST1, which regulates plasma membrane Na^+^/H^+^ antiporters [82], or FBX176 in soybean, which regulates ABA-mediated response to salt stress [83].

Ion transport is the most direct countermeasure to salt stress, since this process is used by plant cells to extrude overabundant ions back to the extracellular environment or retain them by vacuolar internalization [84]. Regarding transporters, there are two DEGs here: ABC transporter (Vitvi11g01198) and NTR/PTR (Vitvi17g00530). Both belong to large families of transporters with a broad range of possible substrates including inorganic cations [85,86]. Overexpression of certain ABC transporters in Arabidopsis (ABCG36) have shown increased salt and drought tolerance [87]. On the other side, recent research demonstrated that two NTR/PTR transporters in grapevine possess Cl^−^ transport capacity (despite being named as “Nitrate/Peptide” transporters), these are VvNPF2.1 (VIT_06s0004g03520) and VvNPF2.2 (VIT_06s0004g03530), which, in the epidermal cells of grapevine roots, take part in Cl^−^ excretion and regulate xylem Cl^−^ loading, preventing the uptake of excessive Cl^−^ anions to the aerial parts [88]. Thus, given its differential expression to salinity, AS1B’s Vitvi17g00530 could also be evaluated as another potential candidate gene for as well for the investigation of Cl^−^ permeability, especially considering that grapevines are generally susceptible to accumulate more Cl^−^ than Na^+^, and, thus, the anion is considered to be a larger contributor to salt toxicity [89].

As it remains to be demonstrated whether and how these grapevine genes take part in salt-detection processes, AS1B could be an appropriate model for its investigation due to its salt tolerance and early differential expression.

The three DEGs from Richter 110 here are Ribosomal protein s12 (Vitvi00g00973), uncharacterized protein (Vitvi10g02353), and carbonic anhydrase (Vitvi17g00819). Carbonic anhydrases have been liked to abiotic stresses, where their activity is mostly known for regulating CO_2_ assimilation in leaves [90]. The implications of this enzyme expressed in the roots are mostly related to nitrogen fixation in legumes [91]; however, its functionality in grapevines experiencing salinity is unclear.

#### 4.3.2. mM-0 d vs. 50 mM-1 d

In this comparison, two DEGs coding for auxin-signaling proteins are present in Vitvi11g01186 and Vitvi07g01071. Root auxin-mediated processes are known to occur in response to salinity [92], and exogenous application of auxin has also been demonstrated to relieve salt damage in crops such as maize [93,94]. Based on this result, a further assessment of the auxin pathways in AS1B and other *Vitis* genotypes would be of interest, to determine if auxin signaling is also a key component to salt tolerance in grapevines. Other transcriptomic studies have also documented auxin-related proteins in grapevines under salinity [61]. Furthermore, a receptor-like protein kinase FERONIA (Vitvi14g02928) is also a DEG here, which could be associated with auxin-promoted root development, as documented in Arabidopsis [95].

Cell-wall-related DEGs appear in this category, such as endoglucanase Vitvi07g00352, extensin Vitvi18g02848. and xyloglucan endotransglucosylase Vitvi08g01617. These proteins participate in the restructuring process of cell walls, maintaining the mechanical integrity of tissues against stresses [96].

### 4.4. Functional Characterization of Genes with a Continuous Differential Expression from Comparison 3 (50 mM-0 d vs. 50 mM-7 d) until Comparison 6 (50 mM-0 d vs. 85 mM-7 d)

#### 4.4.1. Ion Transport

AS1B showed two NRT/PTR DEGs (LOC100258695 and LOC100259122) (Figure 6 and Supplementary Data S4). Relative expression of LOC100258695 remains higher in Richter 110 than in AS1B across all treatments (Supplementary Figure S1), while, in LOC100259122, relative expression earlier in AS1B becomes significantly lower than in Richter 110 at 50 mM-7 d and 85 mM-1 d (Supplementary Figure S2). In the same way as the NTR-PTR proteins are differentially expressed at 50 mM-0 d vs. 50 mM-1 d (Section 4.3.1), it would be worth further investigation into whether the downregulation of these particular NRT/PTR could also lead to lower Cl^−^ uptake to the shoots.

Richter 110 differentially expressed only cation/H^+^ antiporter (LOC100264818) (Figure 6 and Supplementary Data S4), and its relative expression was higher than that of AS1B from 50 mM-0 d up to 85 mM-7 d (Supplementary Figure S3). Generally, cation/H^+^ antiporters are involved in vacuolar cation internalization, releasing protons to the cytosol, while also introducing cations in the tonoplast [97]. Their activity is usually coordinated with proton ATPases, since these employ ATP energy to pump protons back inside the tonoplast, balancing the cytosolic pH decrease from the activity of Cation/ H^+^ antiporters [98]; however, no DEG-coding-proton ATPase has been found in this comparison. LOC100264818 does not belong to the CPA1 group involved in Na^+^ uptake in grapevine [99]. Phylogenetic studies suggest the Cation/H^+^ antiporter coded by LOC100264818 is not closely related to Na/H^+^ antiporters; instead, its nearest homolog in Arabidopsis is CHX20, a K^+^/H^+^ antiporter localized in the endomembranes, with a possible role in osmoregulation [100,101].

Heavy metal transport is often associated with salt stress, as saline soils are often found to be polluted by heavy metals. When these elements reach toxic concentration in plant tissues, they can have an inhibitory effect on enzymes; however, as oligo nutrients, they are essential for electron transport and the catalytic activity of some ROS scavenging enzymes [102]. AS1B and R110 each differentially expressed two nodulin-like proteins. (LOC100259740 from AS1B, LOC100244140 from R110, and LOC100249526 from both) (Figure 6 and Supplementary Data S4). In non-nodulating plants, nodulin homologs are a family of transporters for a broad range of substrates, i.e., in grapevine nodulin-like proteins, they have been reported to transport iron [103].

#### 4.4.2. Secondary Metabolism

One of the main roles of secondary metabolism is the removal of reactive oxygen species (ROS) [104]. Oxidative damage is caused by an excess of reactive oxygen species. Under salt stress, ROS activity is increased, mainly because of ionic disruption at electron transport chains and, in the aerial parts, oxygen accumulation due to stomatal closure [105]. Peroxidase enzymes and several secondary metabolites, such as flavonoids and phenolic compounds, can reduce ROS into less harmful products [106]. This category holds the most unpaired results between both varieties, and genes related to secondary metabolism have a greater presence in R110 than in AS1B, with 29.23% (20 DEGs) from R110 against 20% (9 DEGs) from AS1B (Figure 6).

Expression of secondary metabolism genes could be correlated to either prevention or recovery from oxidative damage [107]; we did not perform any direct ROS measurement, e.g., histochemical staining with nitroblue tetrazolium and diaminobenzidine tetrahydrochloride [108] to determine oxidative damage. Nevertheless, earlier tissue decay and mortality is closely associated with oxidative damage; thus, it could be suggested that this is the case for R110 at late stages of treatment. AS1B had greater expression of secondary metabolism genes in early stages, at 50 mM day 0 and day 7 (Figure 3). An alternate hypothesis could be given to explain these transcriptomic results. For example, AS1B could have either suffered initial oxidative damage due to ion toxicity, but it was eventually stabilized by ion extrusion, or could be able to accumulate antioxidative compounds at an early stage and protect itself when ROS were not accumulated yet. In contrast, R110 may have received more oxidative damage than AS1B in the later stages of saline irrigation, either due to a less efficient ion-extrusion activity or a less sensitive response at earlier stages of the salt treatment.

#### 4.4.3. Mechanical Adjustment: Cell Wall and Cytoskeleton Dynamics

Differentially expressed gene LOC100244452, coding actin, is shared in common between both varieties; actin motility is essential for cytoskeleton reorganization in root cells, which accelerates elongation during water stress [109]. R110 also expressed a DEG for LIM-domain-containing protein WLIM1 (LOC100253922), which binds to actin and enhances its activity. Eight (17.78%) cell-wall-related genes differentially expressed by AS1B code the following: one dirigent protein (LOC100261265), two arabinogalactan proteins (LOC100249036 and LOC100267709), three pectin esterase inhibitors (LOC100250066, LOC100255225 and LOC100257235), and two xyloglucan endotransglucosilase/hydrolase (LOC100250908 and LOC100257235) (Figure 6 and Supplementary Data S4). R110, on the other side, expressed nine genes (13.85%) in this category: three dirigent proteins (LOC100244918, LOC100253541 and LOC100263839), one xyloglucan endotransglucosilase/hydrolase (LOC100852777), 1 pectinesterase inhibitor LOC100251832), two arabinogalactan proteins (LOC100248693 and LOC100267709), one beta-glucosidase (LOC100245197), and one BURP domain protein (Vitvi04g01866) (Figure 6 and Supplementary Data S4). Dirigent proteins catalyze the coupling of monolignols to build lignin complexes [110]. Lignin increases the rigidity and mechanical resistance of cell walls and reinforces the cohesiveness between cells; under salt-stress conditions, this could be important to endure the structural tissue deformation caused by turgor loss and also to enhance root elongation, as they harden to break through the soil [111]. Arabinogalactans proteins are involved in the maturation of the sclerenchyma cells, promoting the development of their heavily lignified secondary cell wall [112], and, for this reason, sclerenchyma is a rigid tissue that surrounds xylem vessels and cortical regions and supports them with additional mechanical resistance, which is important to ensure hydraulic conductivity and prevent cavitation and embolism under water stress [50]. Not only cell walls reinforce, but also splits and reshapes (a process known as cell wall remodeling), to allow tissue growth and repair, especially to maintain root elongation. Grapevine roots are reported to favor growth under drought conditions, toward soils with better water availability, while reducing growth in aerial parts [51]. To promote cell wall remodeling, extensins and beta glucosidases, especially xyloglucan endotransglucosylase/hydrolase, are required. Xyloglucan endotransglucosylase/hydrolase has both the capacity to hydrolyze and attach xyloglucan units [96]. Pectin esterification leads to its deposit on the cell walls; as pectin esterase inhibitor suppresses the activity of pectin esterases, it promotes the release of pectin homogalacturonids, which are essential for cell wall rebuilding and cell cohesion [113]. Finally, the BURP domain has been identified in several apoplast proteins related to cell wall relaxation and enlargement [114].

AS1B had a higher percentage of DEGs dedicated to cell wall dynamics overall, but the total amounts of DEGs were superior in R110. Proportionally, AS1B favored cell wall remodeling (five DEGs) against reinforcing (three DEGs), while the opposite is observed in R110 (four DEGs for remodeling and five DEGs for reinforcing), but whether this has an influence on survival rates under salt stress remains unclear. Despite the documented importance of cell wall integrity as a main mechanical support for plant tissues under salt stress, our results cannot confidently relate the survival differences seen for both accessions and, particularly, the earlier tissue decay from R110, to their expression profile in cell wall dynamics. Nevertheless, cell walls are one of the main targets for oxidation by ROS and ion toxicity [115]; therefore, despite R110 and AS1B having similar levels of DEGs related to cell wall dynamics, their cell wall stability may still be influenced by the differences in their secondary metabolism DEGs.

#### 4.4.4. Hydric Regulation

Genes for hydric regulation were one the most unpaired functional categories in our results: R110 surpassed AS1B with eight genes against four, which is equivalent to 12.31% against 8.89% of their respective numbers of core DEGs. We considered hydric regulation DEGs as those which have countermeasures for water stress directly, by promoting mechanisms for water retention and intake, without taking part in ion transport. AS1B expressed a late embryogenesis abundant protein (LOC100244040), two aquaporins (LOC100240701 and LOC100233027), and one osmotin (LOC100263272) (Figure 6 and Supplementary Data S4). R110 expressed three aquaporins (LOC100233002, LOC100250080, and LOC100252631), three osmotin/thaumatins (LOC100242737, LOC100246096, and LOC100259841), a vicilin-like (LOC100256465), and a polyol transporter (LOC100852699) (Figure 6 and Supplementary Data S4).

Aquaporins are transmembrane channels with passive water-transport activity; in plant cells, they are present in both the cellular membrane and vacuolar membrane. They are essential for the plant´s regular water intake [116], and their importance in hydric regulation has been extensively studied in grapevines for drought stress [48,49,116,117,118]. In fact, Richter 110’ s remarkable tolerance to a water deficit has been linked to aquaporin regulation [119]. Aside from water, plant aquaporins were found to be permeable to Na^+^ and K^+,^ when expressed in transgenic Xenopus oocytes [117]; however, the ion exclusion activity of grapevine aquaporins and their implications in salt tolerance remains to be investigated.

Late embryogenesis proteins are abundant in hydrophilic amino acids, which promote cytosolic water retention [120], and they have been found upregulated under salt- and drought-induced water stresses in different grapevine varieties such as Cabernet Sauvignon, Trincadeira, and Touriga Nacional, as well as the rootstock of Couderc [121,122]. Up to 60 genes, encoding LEAs, have been found through the grapevine genome [123]. Vicilin is a sucrose-binding globin protein with water solubility, which contributes to the osmoprotectants with sucrose and also act as such itself [123]. Polyols such as glycerol, mannitol, or sorbitol are organic osmoprotectans, which are able to balance the unpaired water potential caused by high extracellular ion concentration, lessening the water loss by osmosis. Osmotin has been found to increase relative hydric content and water deficit tolerance when overexpressed in transgenic tobacco and tomato, possibly by an accumulation of proline and glycine betaine, which act as osmoprotectants as well. It also contributes to H_2_O_2_ removal by stimulating peroxidase and superoxide dismutase [124]. R110 appears to show a broader response to water stress than AS1B, by expressing more hydric-regulation DEGs, which may suggest that the survival advantage of the wild grapevine under salinity might come from other mechanisms that are less focused on the osmotic stress component in salinity.

#### 4.4.5. Regulatory Systems: Hormone Response and Signaling

AS1B expressed four (8.89%) signaling-related DEGs (Figure 6 and Supplementary Data S4): Plasma membrane-associated cation-binding protein 1 (LOC100261607) interacts with calmodulin in Ca^+^-mediated processes, including vacuolar internalization and ion extrusion [125]. IQ-domain protein (LOC100255106) interacts with calmodulin and myosin as well. Moreover, 3-oxo-Delta(4,5)-steroid 5-beta-reductase (LOC100264565) is involved in phytosteroid synthesis and has been associated with vascular development in Arabidopsis [103]. MARD1 (LOC100245904) participates in the SnRK1, which regulates carbohydrate and energy metabolism, and promotes ion transport through the SOS complex (also regulated by calcium–calmodulin) [79].

R110 expressed seven (10.77%) signaling-related DEGs (Figure 6 and Supplementary Data S4): Phosphatase inhibitor (LOC100244758), which inhibits phosphatases and promotes the phosphorylation of sugars and kinase signaling activity [84]. Leaf Rust Disease-Resistance Locus Receptor-Like Kinase (LOC100262212) and pathogenesis-related protein STH-21 (LOC100262212) are both known as biotic stress regulators, but their role against salt stress is unclear [126]. Brassinosteroid insensitive 1-associated receptor kinase 1 (LOC100264403) coordinates several brassinazol pathways associated with programmed cell death, pathogen resistance, and salt-stress tolerance [127]. G-type lectin S-receptor-like serine/threonine-protein kinase (LOC104878665) is linked to higher chlorophyll content in aerial tissues and lower ion leakage in salt-stressed Arabidopsis [77]. Aminotransferase ALD1 (LOC100253830) and ser/thr kinase PBL2 (Vitvi16g02090) coordinate systemic acquired resistance against microbial pathogens [128,129].

Regarding hormone response, R110 expressed four DEGs (Figure 6 and Supplementary Data S4): Ethylene-responsive transcription factor ERF098-like (LOC100263477). Salicylate carboxyl methyltransferase (LOC100253073) participates in the synthesis of salicylic acid; this hormone has been reported to lessen growth inhibition and tissue necrosis under salt stress [130]. Pathogenesis-related proteins LOC100246525 and LOC100246525 have been reported to interact with cytokinin and ABA, and their low cytokinin and high ABA levels stimulate ion transport through the SOS pathways [131]. In the case of AS1B (Figure 6 and Supplementary Data S4), the hormone-response DEGs included allene oxide synthase (LOC100252012) and *Pyricularia Oryzae* Resistance 21-like (LOC100852467). Allene oxide takes part in the biosynthesis of jasmonic acid, which is to be reported to be increased under salt-stress conditions, promoting the expression of peroxidases and osmotin [132]. *Pyricularia Oryzae* Resistance 21 is a glycoprotein reported to be able to decrease salicylic acid activity, while promoting jasmonic acid and ABA during pathogen defensive responses in rice [133]; however it is unclear whether the grapevine homolog here shares similar functions. Despite the hormone response in both varieties sharing similarities in terms of salicylic acid and ABA signaling, which is known to promote salt tolerance in grapevine [134], the signaling processes from AS1B could be more inclined toward ion transport through the calcium-mediated pathways, while R110 seems to maintain a more diverse regulatory activity (with a larger amount of DEGs) but is less specific toward salt stress, with responses against biotic stresses aside from salt stress.

#### 4.4.6. Biotic Stress

Responses to abiotic and biotic stresses are known to overlap in some proportion, especially in traits like cell wall reinforcing and secondary metabolism, which may help in the defense against pathogens and herbivores, while detoxifying ROS caused by abiotic stresses such as [135,136]. Nonetheless, in grapevine rootstocks, this association seems less relevant to salt tolerance, as more specific responses toward salt exclusion are still necessary. Rootstock varieties with poor salt-exclusion capacity, such as K51-40 or 101-40 [137], still present a range in disease resistance [138]; yet this is not absent in proficient salt-excluding rootstocks such as Ramsey [139]. AS1B presented three DEGs with a specific response to biotic stress, while R110 presented five. In relation to the results for the DEGs for the regulatory processes, R110 seems to be dedicating a bigger portion than AS1B of the hormonal and signaling pathways for biotic stresses, rather than for salt stress (Figure 6 and Supplementary Data S4).

Although the experiment was carried out in non-sterile conditions, we did not observe any significant symptoms of pathogenicity affecting the grapevines; therefore, as also suggested by the DEGs related to signaling processes, we might consider R110 to be producing higher levels than AS1B of a non-specific response against salt stress, which in this case is the response against biotic stress.

#### 4.4.7. Post-Translational: Protein Stabilization and Ubiquitin-Mediated Degradation

Ubiquitins, heat-shock proteins, and proteases function as post-translational protein modifiers, usually by the hydrolysis of either specific sites or the whole protein. In contrast, chaperones act as post-translational stabilizers [140]. Under environmental stress, they are known to regulate signaling protein and transcription factors in Arabidopsis, potato, and maize, also serving as quality control systems by the degradation of misfolded proteins [141,142]. However, in grapevine, their implications in salt damage have not been studied. In this category, AS1B show two DEGs (4.44%) (Figure 6 and Supplementary Data S4), subtilisin-like protease (LOC100245280) and chaperone dnaJ11 (LOC100245280), while R110 only expressed one (1.54%), aspartyl protease (LOC100265510), which is a ubiquitin-mediated protease [143].

Defective misfolded proteins could be one of the consequences of excessive ion leakage into the cytoplasm, although oxidative agents would cause damage to the protein as well [144]. Both AS1B and R110 expressed one protease associated with the removal of misfolded proteins; however, the key difference in this category would be that AS1B also expressed a chaperone, which prevents protein misfolding at the translational level Therefore, post-translational processes could be worth investigating for their potential implications in salt tolerance. Since translational and post-translational efficiency cannot be directly estimated from RNAseq data, there would be a proportion of futile DEGs (those that code proteins that eventually failed to achieve a function), for each accession, to be additionally considered along with the transcriptomic differences between those we are assessing in both accessions, and, according to the results in this category, this proportion would be larger in R110.

## 5. Conclusions

Wild grapevines possess adaptations to environmental characteristics of their habitat, developed through natural selection. AS1B is a wild grapevine that possesses a remarkable capability to endure salinity; hence, its biological mechanisms are worth disentangling in order to expand the knowledge on grapevine salt tolerance and, consequently, improve breeding strategies for salt-tolerant varieties. In the present study, we analyzed the transcriptomic profile of AS1B when facing salinity, aiming to understand the genetic component being expressed in AS1B’s response to salinity. We compared the transcriptomic profile of AS1B with the widely cultivated rootstock Richter 110, which is a not a salt-tolerant variety, and, thus, found key transcriptional differences that could deliver hints about AS1B’s advantage regarding salt tolerance.

**Figure 7:**
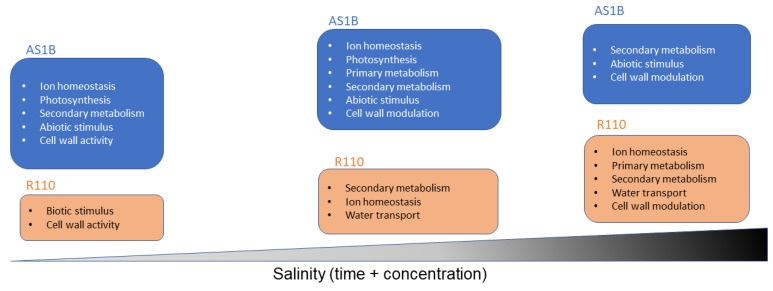
General transcriptomic profiles between AS1B and Richter 110 over increasing salt concentration and exposure time.

In summary, our results suggest that the advantage of AS1B in surviving under severe salt stress could reside in its mechanisms of salt tolerance being activated at earlier stages of salinity, which also keep pace as this condition increases. This is reflected by the changes in AS1B’s gene expression observed at the first two instances when the concentration of salt treatment increased, while Richter 110 kept a constitutive expression. Furthermore, differences between both varieties were still noted across higher stages of salinity and different exposure times, where AS1B displayed a more specific response to salinity compared to Richter 110 (Figure 7).

AS1B’s adaptative strategy toward incremental salinity could be a contrasting addition to the known attributes in commercial salt-tolerant rootstocks, such as 140 Ruggeri and Ramsey, which sustain a constitutive gene expression against salinity, hence expanding the possibilities for understanding different mechanisms of salt tolerance for choosing suitable traits for breeding.

Our transcriptomic findings will guide further physiological studies on AS1B, to confirm its salt-tolerance capabilities and reassess its value for the development of salt-tolerance varieties. Additionally, given the increasing accessibility of sequencing technologies and the pipelines for whole genome assembly and annotation, the specific genetic information from AS1B could be worth revealing in future studies. Our study suggests that AS1B would be a valuable candidate to assist with the development of salt-resistant rootstocks toward climate change. Nevertheless, AS1B is an example of still extant wild-grapevine communities that have naturally adapted to abiotic stresses, demonstrating the value of natural reservoirs for the search of characteristics that could help the grape and wine industry to face future environmental challenges.

## Data Availability

https://www.ebi.ac.uk/ena/browser/view/PRJEB48962 (accessed on 18 April 2022).

## Supplementary Materials

The following supporting information can be downloaded at: https://www.mdpi.com/article/10.3390/plants11202688/s1. Supplementary Data S1: Total number of different expressed genes in different treatment conditions and control in Richter 110 and AS1B genotypes. Supplementary Data S2: Number of transcripts with change expression level in each treatment and control group, which includes upregulated and downregulated transcripts in Richter 110 and AS1B genotypes. Supplementary Data S3: List of genes different expressed in the control and treatment conditions in Richter 110 and AS1B and the distribution of differentially expressed genes into functional categories in both accessions. Supplementary Data S4: List of core genes in Richter and AS1B genotypes and the distribution of differentially expressed genes into functional categories in both accessions. Supplementary Data S5: Functional annotation of DEGs at Control vs. 50 mM-0 d vs. 50 mM-1 d. Supplementary Data S6: Primers designed for the RT-qPCR. Supplementary Figure S1: Relative expression of LOC100258695 in Richter 110 and AS1B genotypes. Supplementary Figure S2: Relative expression of LOC100259122 in Richter 110 and AS1B genotypes. Supplementary Figure S3: Relative expression of LOC100264818 in Richter 110 and AS1B genotypes. Supplementary Figure S4: Validation of expression profiles of salinity related genes by qPCR. LFC values of qPCR normalized genes were normalized to the reference genes GADPH and actin and to the 0h control for 3g0 treatment. For the rest of the treatments (3g1, 3g7, 5g0, 5g1, and 5g7), the relative control was 3g0. Supplementary Figure S5: Linear regresion of significant regulated RNAseq LFC and qPCR LFC values.

## Abbreviations

**50 mM-0 d**	50 millimolar of sodium chloride per liter of irrigation solution at day 0
**50 mM-1 d**	50 millimolar of sodium chloride per liter of irrigation solution at day 1
**50 mM-7 d**	50 millimolar of sodium chloride per liter of irrigation solution at day 7
**85 mM-0 d**	85 millimolar of sodium chloride per liter of irrigation solution at day 0
**85 mM-1 d**	85 millimolar of sodium chloride per liter of irrigation solution at day 1
**85 mM-7 d**	85 millimolar of sodium chloride per liter of irrigation solution at day 7
**DEA**	Differential expression analysis
**DEG**	Differentially expressed gene
**GO**	Gene ontology
**R110**	Richter 110
**RNA-seq**	RNA sequencing
