# Supplementary figures and images for: Coastal Wild Grapevine Accession (Vitis vinifera L. ssp. sylvestris) Shows Distinct Late and Early Transcriptome Changes under Salt Stress in Comparison to Commercial Rootstock Richter 110
Source: Plants (Basel). 2022 Oct 12;11(20):2688. doi: 10.3390/plants11202688 (PMC9610063; doi:10.3390/plants11202688)

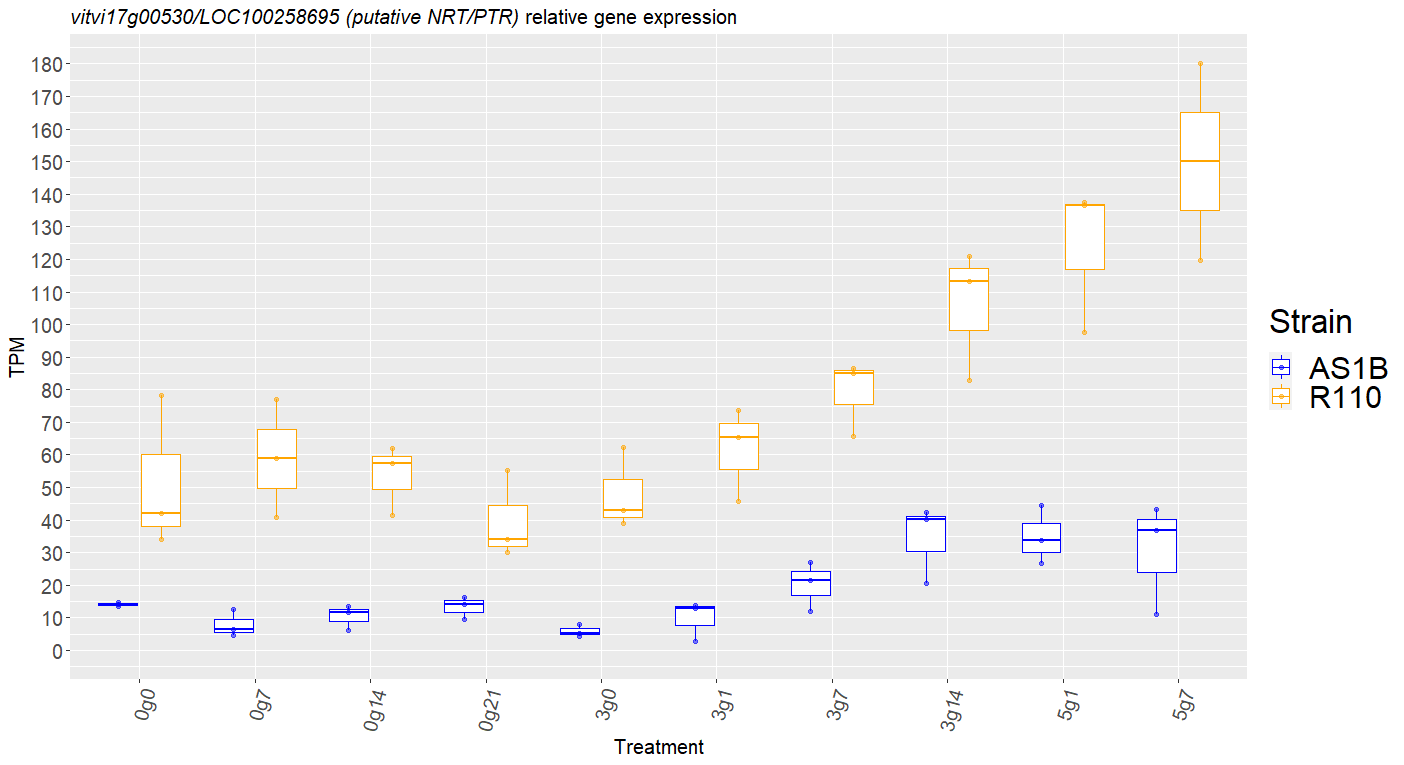

Supplement: Supplementary file 1 [file plants-11-02688-s001.zip › Supplementary Figure S1.png]

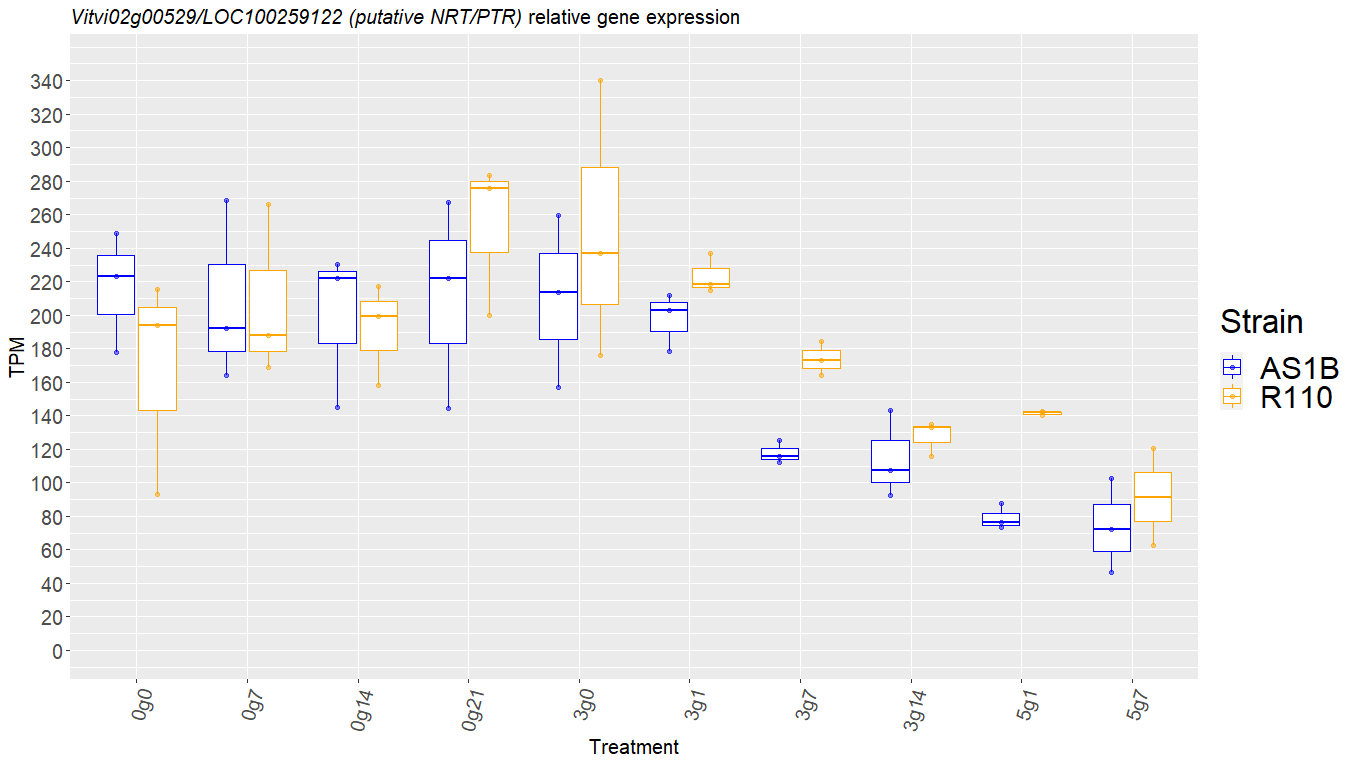

Supplement: Supplementary file 1 [file plants-11-02688-s001.zip › Supplementary Figure S2.png]

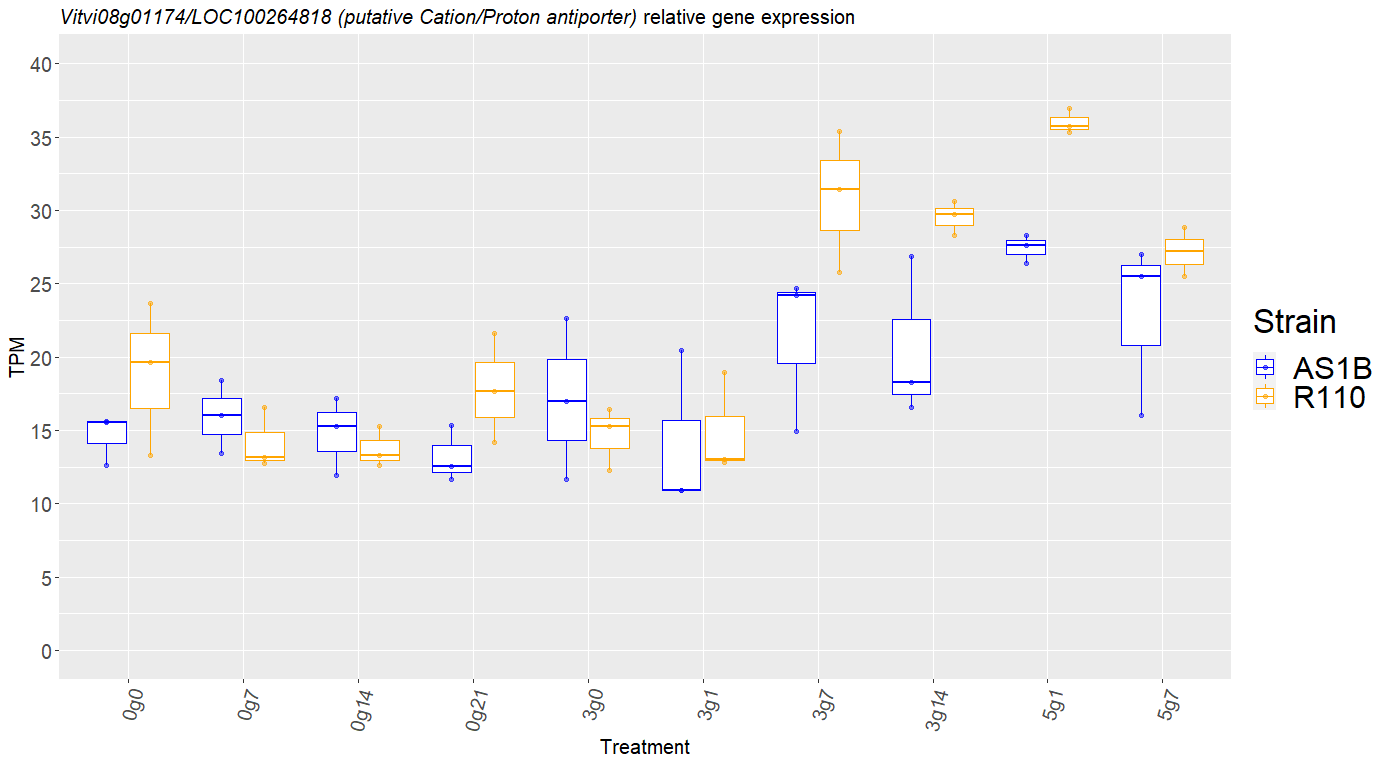

Supplement: Supplementary file 1 [file plants-11-02688-s001.zip › Supplementary Figure S3.png]

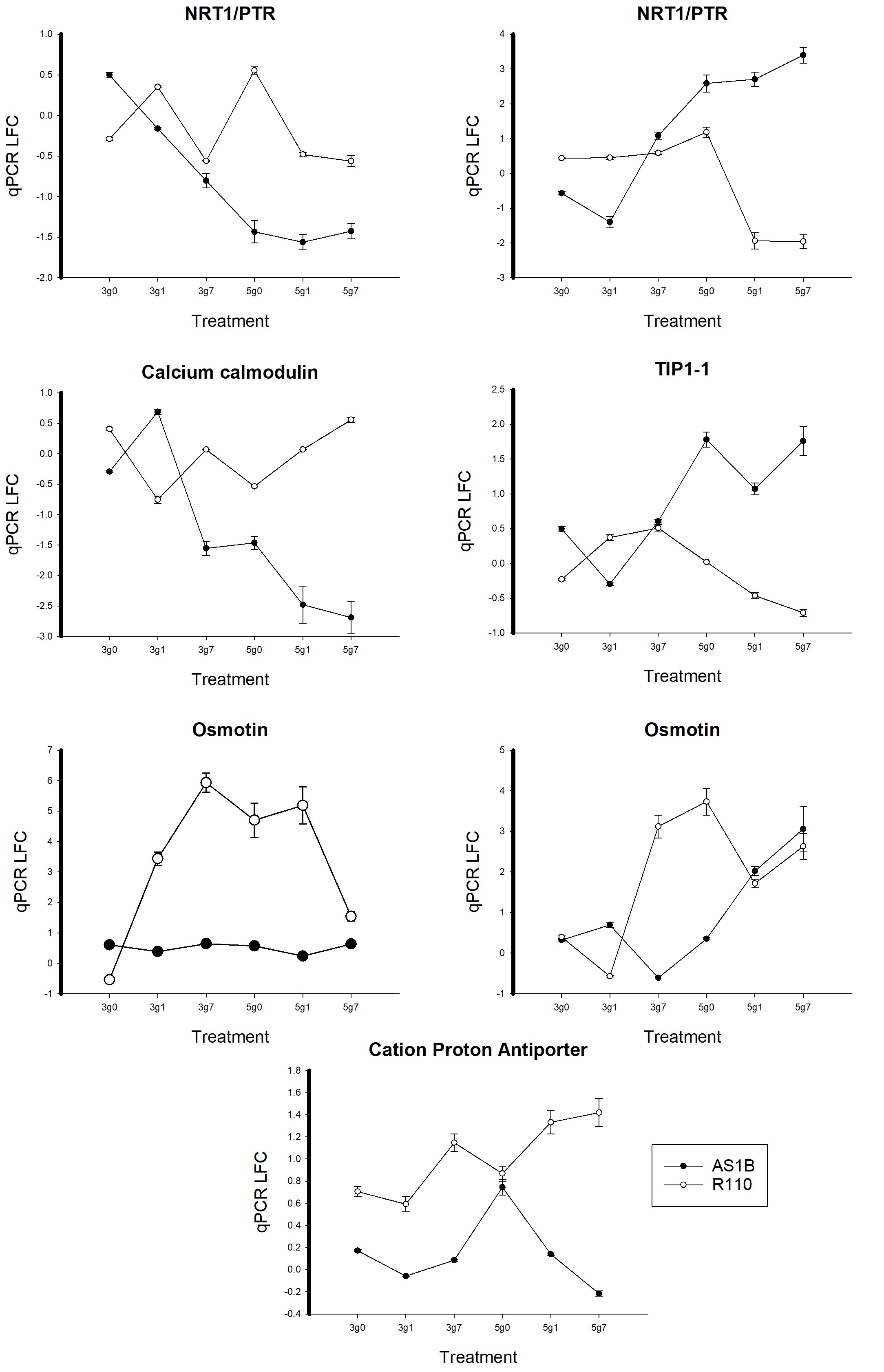

Supplement: Supplementary file 1 [file plants-11-02688-s001.zip › Supplementary Figure S4.jpg]

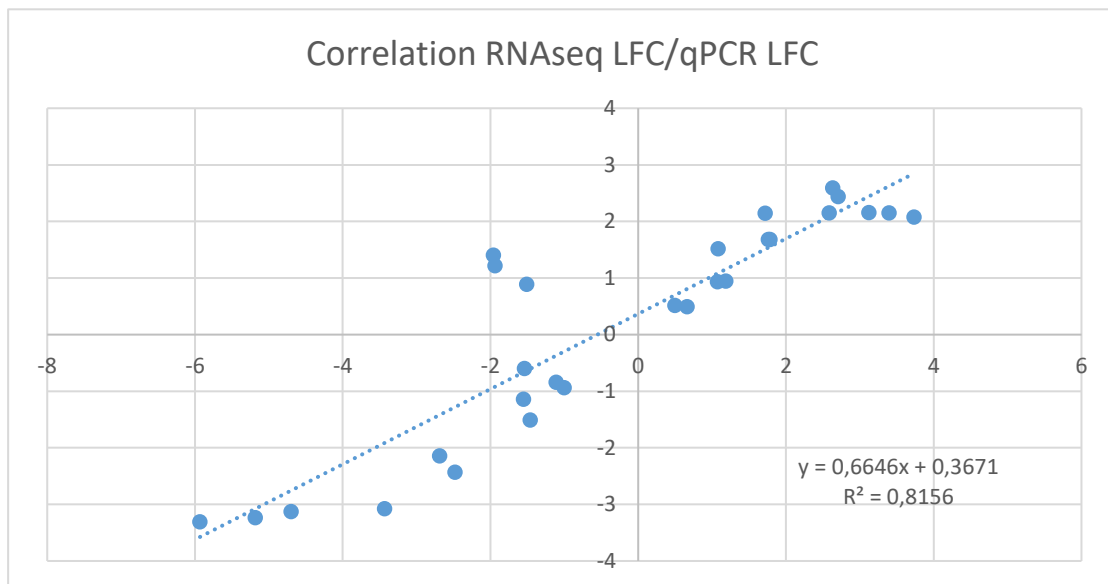

#### Log Fold Chage (FLC) Values

| qPCR       | RNAseq     |
|------------|------------|
| 0,4945447  | 0,51485755 |
| 1,78163725 | 1,67781454 |
| 1,07207576 | 0,92989892 |
| 1,76023676 | 1,67781454 |
| 1,08244696 | 1,5133128  |
| 2,58614946 | 2,14716508 |
| 2,70532716 | 2,43452026 |
| 3,3964267  | 2,14716508 |
| 0,66011261 | 0,49243645 |
| -1,5097225 | 0,88765226 |
| -1,1127685 | -0,8429262 |
| -1,5537486 | -1,1438685 |
| -1,4641236 | -1,5133128 |
| -2,4796051 | -2,4345203 |
| -2,6905592 | -2,1471651 |
| -3,4358647 | -3,0807733 |
| -5,9323514 | -3,3096761 |
| -4,7017413 | -3,1292279 |
| -5,1867175 | -3,2364141 |
| -1,5408891 | -0,6030048 |
| 3,1205225  | 2,15635846 |
| 3,73414294 | 2,07561605 |
| 1,71684241 | 2,14536369 |
| 2,63096398 | 2,59193788 |
| 1,18356955 | 0,94263746 |
| -1,9386567 | 1,21602049 |
| -1,9591089 | 1,40423293 |
| -1,0033678 | -0,9406918 |

Supplement: Supplementary file 1 [file plants-11-02688-s001.zip › Supplementary Figure S5.pdf]
